# Application of lamina replantation with ARCH plate fixation in thoracic and lumbar intraspinal tumors

**DOI:** 10.3892/etm.2013.1170

**Published:** 2013-06-20

**Authors:** DONG ZHOU, LU-MING NONG, GONG-MIN GAO, YU-QIN JIANG, NAN-WEI XU

**Affiliations:** Department of Orthopedics, The Second People’s Hospital of Changzhou, Nanjing Medical University, Changzhou, Jiangsu 213000, P.R. China

**Keywords:** laminoplasty, intradural tumor, internal fixation

## Abstract

The aim of the present study was to investigate the clinical effects of lamina replantation with ARCH plate fixation on patients with thoracic and lumbar intraspinal tumors, following laminectomy. Thirteen patients with thoracic and lumbar intraspinal tumors underwent total lamina replantation with ARCH plate fixation and repair of the supraspinous ligaments, following laminectomy and tumor enucleation. To investigate the clinical effect of lamina replantation with ARCH plate fixation, pre- and postoperative visual analog scale (VAS), and Oswestry Disability Index (ODI) scores were determined, and pre- and postoperative X-ray and magnetic resonance imaging (MRI) examinations were conducted. Computed tomography (CT) examinations were also included in the follow-up. No complications were observed pre- or postoperatively. The VAS and ODI results 2 weeks following surgery and at the final follow-up examination demonstrated a significant improvement compared with the corresponding preoperative results. The X-ray examination results indicated a satisfactory internal fixation location, without any characteristics of a fracture, lumbar scoliosis, kyphosis or instability. Following the surgery, the CT and MRI examination results demonstrated that healing of the lamina bone and repair of the supraspinous ligament had occurred without tumor recurrence or spinal epidural scar recompression. Two of the 13 cases were lost to follow-up. The results indicated that in patients with thoracic and lumbar intraspinal tumors, lamina replantation with ARCH plate fixation following total laminectomy is effective and provides thoracolumbar stability. Furthermore, this has been identified to be an effective technique for preventing intraspinal scar proliferation.

## Introduction

The two basic principles in spine and spinal cord surgery are the complete resection of the intraspinal tumor and the restoration of spinal stability (1,2). Multisegment laminectomy is typically performed to achieve complete exposure and resection of the intradural tumor. However, intraspinal epidural scar adhesions, spinal instability, kyphosis and additional complications are usually identified postoperatively (3,4). Following the introduction of lamina replantation by Raimondi in 1976 (5), various technologies that assist in the procedure have been developed (6–9). The clinical effect of lamina replantation is limited due to a lack of appropriate internal fixation techniques. Although lamina replantation with fixation by a mini-plate is more commonly utilized in cervical treatment, this approach has also been used in thoracolumbar spinal surgery (10). However, further clinical cases and studies of the long-term effects of the method are required. The present study aimed to investigate the clinical effect of lamina replantation with ARCH plate fixation on patients diagnosed with thoracic and lumbar intraspinal tumors, following laminectomy.

## Materials and methods

### Patient data

From February 2009 to June 2010, 13 patients (32 segments) with thoracic and lumbar intraspinal tumors underwent lamina replantation with fixation using an ARCH plate (Synthes Inc., Wilmington, DE, USA) following laminectomy (Table I). Following the surgery, the majority of the patients experienced pain in the thoracic and lumbar regions during rest or when sleeping, along with lower limb numbness and weakness. In addition, the patients endured difficulties associated with urination or defecation. This study was conducted in accordance with the Declaration of Helsinki, and with approval from the ethics committee of The Second People’s Hospital of Changzhou, Nanjing Medical University (Changzhou, China). Written informed consent was obtained from all participants.

### Surgical technique

The patients were administered general anesthesia through tracheal intubation, and the surgery was performed with patients in the prone position. A medial longitudinal incision was made along the thoracic and lumbar spine, and the bilateral sacrospinalis were separated to the small joints. Based on the location of the tumor, which was determined by MRI, the spinous process and vertebral lamina that required cutting were exposed, while the supra- and interspinous ligaments remained intact. The cortex of the lamina was cut (2–3 mm) with a bone drill between the lateral lamina and the inner small joints, and an osteotome was then used to cut through the lamina. The supra- and interspinous ligaments, as well as the ligamentum flavum of the tail were cut in order to isolate the spinous ligament complex. The spinous ligament complex was then turned over and fixed in the head end. The tumor was exposed and removed, which resulted in a reduction in spinal cord compression. The spinous ligament complex was reset, and the inter- and supraspinous ligaments were sutured with polydioxane monofilament synthetic (PDS) II absorbable sutures (DePuy Orthopaedics, Inc., Warsaw, IN, USA). An ARCH steel plate of the appropriate size and shape was inserted and fixed bilaterally with titanium screws, following the resetting of the lamina. The tube placement was drained, and then the incision was washed and closed at each layer.

Antibiotics were routinely administered intra- and postoperatively. On the first day following surgery, the patients were required to perform back muscle exercises in bed. A girdle brace was introduced to patients that performed exercise in bed for three days following the surgery. Two weeks following surgery, the patients were permitted out of bed with the assistance of a girdle brace. The waist brace was removed one month following the surgery.

### Therapeutic evaluations

The visual analog scale (VAS) and the Oswestry Disability Index (ODI) were used for pre- and postoperative therapeutic evaluation.

### Imaging evaluation

To determine the level of internal fixation and spinal stability, lumbar spine X-rays were performed in the posterior-anterior, lateral and flexion-extension positions, one day prior to the surgery, and at 2 weeks, and 1, 3, 6 and 12 months following the surgery. A computed tomography (CT) scan was performed three months following surgery and monthly thereafter, until the lamina bones had fused. Furthermore, the bone growth of the regrafted lamina was evaluated. Six months following the surgery, magnetic resonance imaging (MRI) was performed to detect tumor recurrence and scar oppression in the spinal canal, and the repair of the ligaments.

### Statistical analysis

The VAS and ODI scores were expressed as the mean ± standard deviation. A paired t-test was performed using the SPSS software, version 11.0 (SPSS, Inc., Chicago, IL, USA). P<0.05 and P<0.01 were considered to indicate a statistically significant difference.

## Results

### Clinical effects

The thirteen patients in this study underwent successful surgery and demonstrated primary healing. One patient with ependymoma who developed postoperative cerebral spinal fluid (CSF) leakage spontaneously recovered following conservative treatment. The development of complications following the surgery did not occur in the other patients. In addition, the thoracolumbar and back pain was significantly relieved or disappeared following the surgery, while the numbness of the limbs also decreased in intensity.

One month following the surgery, two of the cases were lost to follow-up. The follow-up period in the remaining 11 patients was 9–22 months, with a final follow-up period of >3 months. The VAS and ODI scores of the patients are shown in Table II.

### Biomechanical test results

In the present study, a biomechanical test of the lumbar lamina replantation with ARCH steel plate fixation was completed, which showed that following the surgery the vertebral strain increased by 36–60%, the transverse transposition increased by 38–49%, the stiffness decreased by 26–57% and the carrying capacity decreased by 32–45%. In addition, lamina replantation with plate fixation resulted in similar levels of vertebral strain, transposition, stiffness and carrying capacity, following surgery, compared with normal levels (difference, 4–11%; P<0.05).

### MRI findings

**Figure 1.**
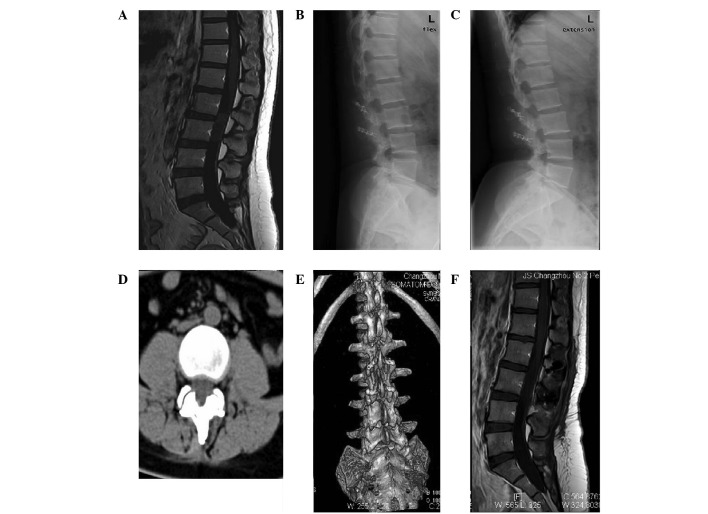
Case 2. Ependymoma at the L2–3 level. (A) Prior to surgery, magnetic resonance imaging (MRI) identified that the L2–3 spinal space was occupied by the tumor. (B and C) One year following the surgery, X-ray examination indicated no fixation transposition or fracture, lumbar instability or kyphosis. Three months following the surgery, favorable lamina bone fusion was indicated by (D) a postoperative computed tomography (CT) scan and (E) 3-D reconstruction. (F) Six months following the surgery, no intraspinal scar adhesions or restenosis were identified by MRI.

Following the surgery, no fixation transposition or fracture, lumbar instability or kyphosis was identified. In patients with a follow-up period of >6 months, favorable healing of the vertebral lamina without bone restenosis or intraspinal epidural scar adhesions was observed (Fig. 1).

## Discussion

It has been indicated that a posterior approach to the spine may provide access to a wider area of the spine during surgery, and therefore reveal the intraspinal tumor for complete resection. However, the clinical application of a posterior approach is limited due to the wide removal of bone and ligaments, as well as the high risk of peridural adhesions and spinal cord injury, which are associated with weak bone protection (11,12).

Previous short-term follow-up studies on the laminectomy procedure demonstrated a high degree of satisfaction and spinal stability (13,14). However, in long-term studies, the satisfaction rate decreased to <60%, as lumbar instability resulted in chronic lower back pain (15,16). A long-term study by Mullin *et al* (17) identified that 54% of patients with dynamic lumbar instability had previously undergone total laminectomy. In addition, Papagelopoulos *et al* (14) revealed that 28% of young patients (≤30 years) treated with thoracolumbar multisegment laminectomy exhibited spinal deformity. Iida *et al* (11) demonstrated that extensive laminectomy induced intervertebral instability. Moreover, a posterior structure resection was more likely than nucleus pulposus removal to cause postoperative spinal instability. However, Tsuji *et al* (18) demonstrated that lower back pain and sensory disturbance were marginally improved following laminectomy.

Laminoplasty is considered to be important for young patients with benign tumors, so as to avoid postoperative complications associated with laminectomy, such as refractory back pain and spinal deformity (5,19). Two-stage antero-posterior spinal fusion and internal fixation may provide favorable clinical outcomes compared with laminectomy; single-stage extensive laminectomy may cause posterior bone deficiency (12). In China, single-stage laminectomy, nail-stick system fixation and bone fusion surgery have been used to achieve immediate spinal stability. These procedures have resulted in a large surgical field, segment movement disorder and peripheral segment degeneration (20).

The long-term postoperative complications have not been increased; however, numerous scholars have adopted various techniques in order to treat thoracic and lumbar intraspinal tumors (2,6–9,13,21,22). Menku *et al* proposed the application of a mini-plate in thoracic and lumbar laminoplasty, as opposed to its traditional usage in the cervix, to fix the replanted lamina, and to provide immediate stability and a smaller surgical incision (10).

Results of biomechanical tests showed that the technique of lamina replantation with ARCH plate fixation was able to improve spinal stability, compressive resistance and anti-bending, -shearing and -rotation abilities.

Numerous studies that aimed to prevent postoperative epidural adhesion, such as by the use of adipose tissue, amniotic membranes, silicon films and silicon rubber sheets, and hormone and anti-inflammatory drug instillation, have not been successful (11,12,24–26). However, we propose that the vertebral plate is an effective and safe isolation method that prevents scar adhesion in lamina replantation fixation.

A previous study revealed that supra- and interspinal ligaments were well innervated, and that this innervation may form the basis of neurological feedback mechanisms for the protection and stability of the spine (27). Studies by Hotta (28) and Newman (29) demonstrated the importance of the supraand interspinal ligaments in the enhancement of spinal flexion stability. In addition, Sano *et al* (30) and Joson *et al* (31) proposed methods of conserving the supraspinous ligament during laminectomy. Hirofuji *et al* (32) suggested reconstruction of the supra- and interspinous ligaments using artificial ligaments. In the present study, unilateral supra- and interspinous ligaments were cut, reversely rotated with the spinous process and the lamina, and repaired with PDS II absorbable sutures following tumor removal and lamina replantation.

Lamina replantation with ARCH plate fixation and ligament repair were adopted in the current study, as the technique allows for smaller surgical incisions, allows bone protecting the spinal cord to be conserved, prevents spinal instability and kyphosis, and preserves the spinous process during lamina replantation, resulting in a favorable appearance. In addition, the restoration of the ligament-nerve-muscle reflex system of the supra- and interspinous ligaments aids lower back movement. Furthermore, the technique prevents epidural adhesion following laminectomy. Moreover, a second surgical procedure is then safer and simpler, as the posterior bony structure is preserved. Lamina replantation enables muscle and soft tissue attachment, increasing postoperative perispinal muscle function. Additionally, the biomechanical environment of the surgical segments is recovered, which prevents movement loss and peripheral segment degeneration.

Thoracolumbar laminoplasty using the posterior approach is beneficial, as it retains the posterior spinal structures and prevents postoperative bleeding, scar adhesions, instability, subluxation and kyphosis. In addition, it provides uncomplicated access when further surgery is required. Lamina replantation with titanium plate fixation has been demonstrated to be a favorable surgical procedure that is not limited by the patient’s age, the surgical site or the number of impaired segments.

## Figures and Tables

**Table I.** Clinical data of patients.

Case no.	Gender	Age (years)	Diagnosis	Position	Replantation segments
1	Male	45	Neurilemmoma	Thoracolumbar segment	2
2	Female	40	Meningioma	Thoracic vertebra	2
3	Female	33	Hemangioma	Thoracic vertebra	2
4	Female	46	Neurilemmoma	Thoracolumbar segment	3
5	Female	28	Astrocytoma	Thoracolumbar segment	3
6	Male	42	Ependymoma	Thoracolumbar vertebra	3
7	Female	48	Meningioma	Thoracic vertebra	2
8	Male	38	Neurilemmoma	Thoracolumbar segment	3
9	Male	52	Meningioma	Lumbar vertebrae	2
10	Male	46	Meningioma	Thoracolumbar segment	3
11	Female	33	Meningioma	Thoracolumbar segment	2
12	Female	36	Ependymoma	Lumbar vertebrae	2
13	Female	42	Neurilemmoma	Thoracolumbar segment	3

**Table II.** VAS and ODI scores.

Scale	One day prior to surgery	Two weeks following surgery	Final follow-up
VAS	8.8±1.5	2.8±1.3^a^	1.1±0.4^a,b^
ODI (%)	89.3±9.2	52.8±6.5^c^	10.8±2.3^a,d^

Scores are mean ± standard deviation.

a P<0.01 vs. preoperative score;

b P<0.05 vs. score 2 weeks following surgery;

c P<0.05 vs. preoperative score;

d P<0.01 vs. score 2 weeks following surgery. VAS, visual analog scale; ODI, Oswestry Disability Index.
